# *Rhynchophorus ferrugineus* larvae: A novel source for combating broad-spectrum bacterial and fungal infections

**DOI:** 10.14202/vetworld.2024.156-170

**Published:** 2024-01-20

**Authors:** Nurdjannah Jane Niode, Billy Johnson Kepel, Sofia Safitri Hessel, Tara Sefanya Kairupan, Trina Ekawati Tallei

**Affiliations:** 1 Department of Dermatology and Venereology, Faculty of Medicine, Sam Ratulangi University, Prof. Dr. R. D. Kandou Hospital Manado, Manado 95115, North Sulawesi, Indonesia; 2 Department of Chemistry, Faculty of Medicine, Sam Ratulangi University, Manado 95115, North Sulawesi, Indonesia; 3 Department of Biotechnology, Indonesia Biodiversity and Biogeography Research Institute (INABIG), Bandung 40132, West Java, Indonesia; 4 Department of Biology, Faculty of Mathematics and Natural Sciences, Sam Ratulangi University, Manado 95115, North Sulawesi, Indonesia; 5 Department of Biology, Faculty of Medicine, Sam Ratulangi University, Manado 95115, North Sulawesi, Indonesia

**Keywords:** antimicrobial, broad-spectrum, larvae, *Rhynchophorus ferrugineus*

## Abstract

Antimicrobial resistance is a growing concern due to the growth of antibiotic-resistant microorganisms, which makes it difficult to treat infection. Due to its broad-spectrum antimicrobial properties against a diverse array of bacteria, both Gram-positive and Gram-negative bacteria, and fungi, *Rhynchophorus ferrugineus* larval antimicrobial peptides (AMPs) have demonstrated potential as antimicrobial agents for the treatment of microbial infections and prevention of antibiotic resistance. This study emphasizes the unexplored mechanisms of action of *R. ferrugineus* larvae against microorganisms. Among the most widely discussed mechanisms is the effect of AMPs in larvae in response to a threat or infection. Modulation of immune-related genes in the intestine and phagocytic capacity of its hemocytes may also affect the antimicrobial activity of *R. ferrugineus* larvae, with an increase in phenoloxidase activity possibly correlated with microbial clearance and survival rates of larvae. The safety and toxicity of *R. ferrugineus* larvae extracts, as well as their long-term efficacy, are also addressed in this paper. The implications of future research are explored in this paper, and it is certain that *R. ferrugineus* larvae have the potential to be developed as a broad-spectrum antimicrobial agent with proper investigation.

## Introduction

Antibiotic resistance has become an increasing concern in the field of medicine. The rise of antibiotic-resistant microorganisms has made the management of infectious diseases more difficult, necessitating the urgent development of new antimicrobial agents [1]. A multitude of bacterial species developed the capacity to withstand their effects before the mass production of antibiotics by humans for the prevention and treatment of infectious diseases [2]. This indicates that antibiotic resistance is an inherent process in nature, resulting from the numerous natural challenges they had to endure [3]. Infections of microorganisms that continue to proliferate instead of being inhibited or eradicated (e.g., by antibiotics or natural selection) pose a significant challenge and may, in some cases, be limited by treatment. Antimicrobial resistance is a pressing global public health concern, resulting in a minimum of 1.27 million fatalities on a global scale in 2019 and being linked to approximately 5 million fatalities [4]. The presence of resistance in bacteria and fungi to even a single type of antibiotic or anti fungal agent is sufficient to pose a considerable health risk, and it is not necessary for them to exhibit resistance against all varieties of these agents [4]. There may be severe consequences if resistance to a single antibiotic is present. However, the prevalence of microbial strains resistant to a wide range of drugs further complicates the treatment. Multiple drug-resistance (MDR) [5] is referred to here. MDR pathogen, also known as “superbugs,” is one of the most significant hazards to global public health and cause millions of deaths each year [6]. When microorganisms that are initially difficult to treat acquire the proper combination of resistance mechanisms, it can render all antibiotics and antifungals ineffective, leading to untreatable infections [4].

Efforts to combat multidrug-resistance microorganisms can, among others, be achieved through the discovery of novel alternative therapies [7]. This has resulted in the development of new sources of antibacterial substances from nature, including insects. Insects can withstand pathogenic microorganisms, in particular bacteria, using antimicrobial peptides (AMPs) [8]. AMPs are predominantly produced in the adipose tissues and hemocytes of insects, contributing significantly to their remarkable versatility and survival capabilities [9]. Peptides are released into the hemolymph because the pattern-recognition receptor recognizes the pathogen [10]. AMPs of insects can be categorized into four primary groups, which are primarily distinguished by their structural characteristics or distinctive sequences. These groups include proline-rich peptides such as apidaecins, drosocins, and lebocins, cysteine-rich peptides such as defensins and drosomycins, alpha-helical peptides such as cecropins and moricins, and glycine-rich peptides/proteins such as attacins and gloverins [11, 12]. Attacins, cecropins, diptericins, drosocins, drosomycins, defensins, metchnikowin, and ponericins have been extensively investigated [13]. The majority of active AMPs consists of short peptides ranging from 20 to 50 residues. These peptides are derived from larger precursor proteins that are initially inactive. In addition to these small peptides, medium-sized antimicrobial proteins, such as gloverins (approximately 14 kDa) and attacins (approximately 20 kDa), also exist [11]. Insect AMPs could potentially act as an alternative treatment for bacterial infections, malignancy, and antibiotic resistance [8].

*Rhynchophorus ferrugineus*, a member of the Coleoptera order and Curculionidae family, is commonly found in Melanesia and Southeast Asia [14]. *R. ferrugineus* has emerged as the predominant pest affecting date palm trees, exerting a substantial influence on the date palm industry. *R. ferrugineus* has emerged as a well-known insect referred to as the red palm weevil (RPW) (Olivier 1790). This impact includes considerable economic losses, expenses incurred for the elimination of infested palm trees, a decrease in property values, and the potential risks and expenses associated with preemptive pesticide treatments aimed at safeguarding palm trees from weevil infestations [15]. Numerous studies regarding the management of *R. ferrugineus* have been conducted, in which antimicrobial molecules of *R. ferrugineus* have the ability to inhibit Gram-negative and Gram-positive bacteria [14–16]. The antimicrobial properties of *R. ferrugineus* eggs, larvae, and adults have been demonstrated that their cuticle extracts possess inhibitory effects on *Beauveria bassiana* and Gram-positive bacterial growth [14]. *R. ferruginous* larvae showed inhibitory effects on many types of microorganisms when intestinal extracts were used. These microorganisms included *Enterococcus faecalis* and *Staphylococcus aureus*, *Escherichia coli* and *Klebsiella* spp., and *Candida albicans* and *Penicillium* spp. [17]. Various families of bactericidal peptides and proteins, namely, defensins, attacins, and cecropins, have been identified in the immunized hemolymph of this weevil, along with odorant-and pheromone-binding proteins previously unknown to serve anti-infectious functions in insects [18]. Studies on the antimicrobial characteristics of the newly produced protein pool, which can be detected in low molecular mass hemolymph fractions, have revealed significant antibacterial efficacy against a diverse range of bacterial strains, including *E. coli*, Pseudomonas spp. OX1, *Bacillus subtilis*, and *Micrococcus luteus* [19]. *R. ferrugineus* haemolymphs contain bioactive components that can potentially be used as antimicrobials.

This review aims to discuss the potential *R. ferrugineus* larvae as a novel broad-spectrum source for combating bacterial and fungal infections.

### *R. ferrugineus* Larvae: Composition and Properties

### Overview of the *R. ferrugineus* beetle and its life cycle

**Figure-1:**
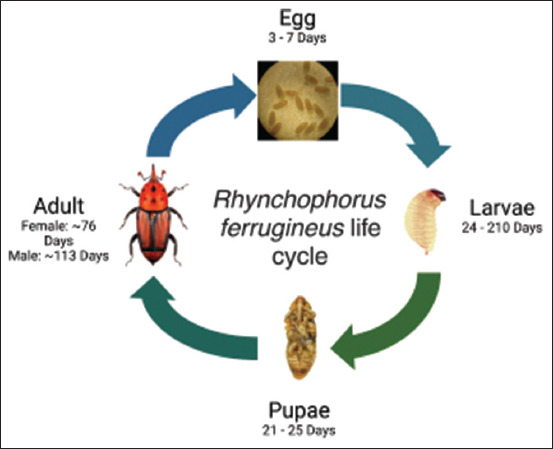
Life cycle of *Rhynchophorus ferrugineus*.

*R. ferrugineus* belongs to the subfamily *Dryophthoridae*, genus *Rhynchophorus* and is one of five species originating from tropical Asia [14, 20]. This pest is sexually dimorphic, which means there are differences between the male and female of this species and has a complete metamorphosis (holometabolous) with four developmental stages (Figure-1)-egg, larva, pupa, and adult - and a life cycle lasting between 45 and 298 days [21, 22].

#### Egg

The *R. ferrugineus* egg is shaped like a cylinder, with rounded edges that is slightly narrowed on the anterior side, and a creamy white and glossy hue, and sizes that range from 0.98 to 2.96 mm [20]. This pest’s eggs take between 3 and 7 days to hatch [23].

#### Larva

*R. ferrugineus* larvae have a creamy white hue, are legless and pyriform, and possess dimensions of a maximum length of 50 mm and a maximum breadth of 20 mm, with a body structure consisting of 13 distinct segments. The coloration of the head capsule ranges from brown to russet-red. In addition, mouthparts exhibit a high degree of chitinization [23]. Larval development spans from 24 to 210 days depending on feeding substrates and temperature [24, 25], whereas the number of larval instars depends on host plant species. *R. ferrugineus* exhibits a tendency toward a decreased duration of development if environmental conditions and the host plant are beneficial to insects. Fertility of female *R. ferrugineus* is influenced by host species [24].

#### Pupae

Pupal development duration can vary from 21 to 25 days [25]. The pupal phase is typically divided into two distinct periods: The pre-pupal period, which spans a duration of 3 days and is characterized by pupae being approximately 25–35 mm in length, and the subsequent pupal period, which lasts 12–20 days and involves color changes in the pupae from cream to brown [26]. As its development progresses, it forms a highly furrowed and reticulated shiny surface [23]. At this stage, the average pupa size is approximately 3×15 mm. *R. ferrugineus* emerges as an adult with a long, curved rostrum that is reddish-brown [26].

#### Adults

Male *R. ferrugineus* specimens were marginally smaller than their female counterparts (approximately 19–42 and 26–40 mm in length, respectively) [20]. Adults possess well-developed wings, allowing them to fly extensively and travel 500–800 m [23]. Adult females produce between 180 and 396 eggs during a 76-day lifespan [21], whereas males live approximately 113 days [26].

### Composition of *R. ferrugineus* Larvae

*R. ferrugineus* is an invertebrate that is consumed in a variety of countries and contains a number of nutritional components. *R. ferrugineus* larvae protein extract showed 58.79% fat, 18.67% carbohydrate, and 15.65% protein [27]. Another study showed that differences in food source of *R. ferrugineus* resulted in different fatty acid compositions, although with similar amounts of total fat and total fatty acids, as well as the same types of fatty acids which were myristic, palmitic, stearic, palmitoleic, oleic, and α-linolenic acids [28]. Kavle *et al*. [29] observed 44 minerals in *R. ferrugineus* larvae, including 11 essential minerals, 29 non-essential minerals, and four heavy metals. *R. ferruginous* larvae also contain an abundance of macro elements such as magnesium, calcium, potassium, sodium, and phosphorus and microelements such as iron, zinc, manganese, and copper. Alkali-soluble, sarcoplasmic, stromal, and myofibrillar proteins and nine essential amino acids [30] were the most abundant types of proteins found in a study.

*R. ferrugineus* larva’s gut is segmented into the foregut, midgut, and hindgut. The fore and hind intestines serve the primary functions of food digestion and fluid absorption, respectively, whereas enzyme secretion and food digestion take place in the midgut [21]. Seman-Kamarulzaman *et al*. [21] found a variety of enzymes in the gut, including protein, hemicellulose, carbohydrate, and antioxidant enzymes such as trypsin aminopeptidase, xylanase, glycosidase, amylase, catalase-peroxidase (PO), and polyphenol oxidase (PPO). In addition, this pest’s gut has its own microbiota of bacteria that are beneficial for their growth and development, as well as their immunity and mating [15, 31]. In a study by Habineza *et al*. [31], the prevalent bacteria (98% of reads) found belonged to Proteobacteria (64.7%), Bacteroidetes (23.6%), and Firmicutes (9.6%) phyla.

The hemolymphs of insects include a diverse array of active substances, such as sugar-binding proteins, lectins, antimicrobial proteins, and peptides, as well as enzymatic systems such as the proPO system. *R. ferrugineus* larval hemolymph comprises an array of proteins, peptides [18], glucose, triglyceride, trehalose, enzymes, lipids, carbohydrates, amino acids, and hemocytes [32–34] that play a crucial role in mediating the host humoral responses [35]. Some of those are important nutritional indices that show the nutrition and metabolic status of these insects [31]. These components play crucial roles in various physiological processes, including immune responses, nutrition, and metabolic status, and contribute to the overall health and functioning of the insect. Knutelski *et al*. [18] identified the bactericidal proteins and peptides (AMPs) in *R. ferrugineus* larval hemolymph, which showed attacin-B, cecropin, defensin, pheromone-and odorant-binding proteins, and one hypothetical AMPs. *R. ferrugineus* larval hemolymph is also rich in enzymes, and some of them are antioxidant enzymes like PPO and PO [36] that may aid in the hemolymph’s antimicrobial activity. Lipases, transaminases such as glutamic oxaloacetic transaminase and glutamine pyruvic transaminase, acid phosphatase, and alkaline phosphatase and acetylcholinesterase, among others [32, 36, 37] are also present in the hemolymph. Amino acids are also important components of the *R. ferrugineus* larval hemolymph, and arginine, histidine, aspartic acid, threonine, tyrosine, alanine, valine, leucine, glutamic phenylalanine, lysine, proline, glycine, methionine, and isoleucine have been identified by Abdel-Razek *et al*. [33]. In addition, the *R. ferrugineus* larval hemolymph contains different types of hemocytes, namely plasmatocytes (approximately 50%), granulocytes (approximately 35%), prohemocytes (approximately 8%), oenocytes (approximately 4%), and spherulocytes (approximately 3%) [33].

### Physicochemical Properties of *R. ferrugineus* Larvae

Kavle *et al*. [27] observed the nutritional and techno-functional composition of *R. ferrugineus* larvae protein extract and found an isoelectric pH (pI) of 4 and a molecular weight range of 10–110 kDa. Techno-functional analysis showed high foaming capacity and emulsion stability with higher heat coagulation at neutral pH (7.0) compared to heat coagulation at pI (4.0) in this study.

Enzymes in *R. ferrugineus* larvae exhibit optimal activity at different pH values. A catalase enzyme found in *R. ferrugineus* was found to have optimal activity at pH 7.5, whereas cellulase and pectinase in the midgut were most stable at pH 4–8 and pH 6–8, respectively [38, 39]. Moreover, compared with highly acidic or alkaline conditions, the catalase enzyme in *R. ferrugineus* was most stable at pH 4–8. *R. ferrugineus* maintains different pH values for different parts of the alimentary canal or digestive tract [40]. *R. ferrugineus’* midgut is divided into different parts at different pH levels: Anterior saccular (pH 5.0–5.4), anterior tubular (pH 4.5–5.0), posterior saccular (pH 5.4–5.6), and posterior tubular (pH 5.0–5.4) parts. The differences in pH conditions are thought to be regulated by a hormone, possibly through stimulating epithelial cells to release secretions of buffering nature into the lumen of the gut [40]. Aljabr *et al*. [41] successfully developed the *R. ferrugineus* midgut cell culture called RPW-1, which had optimal conditions of pH 6.3 and temperature of 27°C (i.e., highest cell density and maximum cell viability).

Studies on the physicochemical properties of the *R. ferrugineus* larval hemolymph are still very limited. Insect hemolymph is the fluid found in insects that is analogous to blood found in most vertebrates, although it does not move within the bounds of vessels; hence, it corresponds to both blood and lymph. Pigments such as carotene, riboflavin, and biliverdin impart a yellow or green hue to the hemolymph of numerous insects. *R. ferrugineus* larvae have a volume of hemolymph of 15% and can be affected by changes in temperature [42].

### *R. ferrugineus* Larvae as a Broad-spectrum Antimicrobial Agent

### Overview of the studies conducted on *R. ferrugineus* larvae as an antimicrobial agent

Because insects lack adaptive immune responses, they generally have good innate immunity. This together with the functions of insect AMPs aids in their successful survival [18]. *R. ferrugineus* is one of the predominant palm plant pests, that is, of interest for its antimicrobial capabilities (Table-1) [14, 16, 18, 19, 43, 44].

**Table-1:** Summary of antimicrobial activity of agents derived from *R. ferrugineus*.

No.	Type of antimicrobial agent from *R. ferrugineus*	Methods of testing	Susceptible microbial strains	References
1	AMP, such as attacin-B-like protein, cecropin-A1-like peptide	Mass spectrometry, sequencing and bioinformatics analysis	Gram-negative strains, specifically *E. coli* C1a and *Pseudomonas* spp. OX1, as well as Gram-positive strains, namely *B. subtilis* ATCC 6051 and *M. luteus* ATCC 4698	[18, 19]
2	Methanol and pentane from *R. ferrugineus* larvae cuticular surface extracts	*In vitro* by testing antimicrobial activity in larvae cuticular surface compound suspension	*B. subtilis, Bacillus thuringiensis, Beauveria bassiana*	[14]
3	Different parts of *R. ferrugineus*’s digestive tract	*In vitro* by utilizing the agar well diffusion method	Gram-positive bacteria (*Enterococcus faecalis* and *Staphylococcus aureus*), Gram-negative bacteria (*E. coli* and *Klebsiella* spp.), *C. albicans*, and *Penicillium* spp	[16]
4	Enzyme phenoloxidase	*In vivo* with immune priming using bacteria and spectrophotometric phenoloxidase assay of collected hemolymph	*E. coli* and *Serratia marcescens*	[43, 44]

*R. ferrugineus=Rhynchophorus ferrugineus, E. coli=Escherichia coli, B. subtilis=Bacillus subtilis, M. luteus=Micrococcus luteus*

A study by Mastore *et al*. [19] revealed that *R. ferrugineus* larvae hemolymph possesses an arsenal of AMPs, that are present in <30 kDa fractions, as might be anticipated from a pest capable of surviving in an environment conducive to bacterial growth. Knutelski *et al*. [18] detected several AMPs in the hemolymphs of *R. ferrugineus* larvae. This pest has demonstrated antimicrobial activity against several bacteria like *E. coli*, *Pseudomonas* spp. OX-I, *B. subtilis*, *M. luteus*, *S. aureus* and *Staphylococcus intermedius* [18, 19, 45].

The currently predominant studies in antimicrobial effects of *R. ferrugineus* are the ones utilizing and observing their gut antimicrobial activity. *R. ferrugineus* has both symbiotic relationships with microbes alongside their antimicrobial activity. The activity and growth of *R. ferrugineus* requires some microbes. The gut microbiota of *R. ferrugineus* exhibits stimulatory effects on the growth and development of this organism, seen by the prolonged development from eggs to prepupae [31].

### *In vitro* studies and *in vivo* studies

Studies on the antimicrobial properties and capabilities of the *R. ferrugineus* larvae have been conducted for both the utilization of this pest for antimicrobial purposes and the study of controlling this pest through information on their microbial resistance and susceptibility [14, 21, 46]. Research by Knutelski *et al*. [18] has provided the identification of AMPs from the immunized hemolymph of *R. ferrugineus* larvae through mass spectrometry, sequencing and bioinformatics analysis. Equally important is that this study also demonstrated AMP activity against infection by several bacterial species. Preliminary results showed ambiguous peaks in the reversed-phase high-pressure liquid chromatography results of the hemolymph components of larvae injected with live *E. coli* and *M. luteus* into their hemocoel. Radial diffusion assay performed using *E. coli* and *S. intermedius* cultures exhibited peaks indicating bactericidal and bacteriostatic activity.

A study by Mastore *et al*. [19] also analyzed the presence of AMPs and antimicrobial properties of *R. ferrugineus* larvae. The larvae were subjected to infection with either inactivated or live bacteria, or a combination of pure lipopolysaccharide and peptidoglycan, to induce the expression of AMPs. The experimental study utilized bacterial cultures of Gram-negative strains, specifically *E. coli* C1a and *Pseudomonas* spp. OX1, as well as Gram-positive strains, namely, *B. subtilis* ATCC 6051 and *M. luteus* ATCC 4698, for immunization and observation of hemolymph antimicrobial activity. Substantial activity against Gram-negative bacteria was observed in immunized larvae hemolymph at both <30 kDa and <10 kDa fractions, seen through 99.9% bacteria mortality. However, this activity in naive larvae hemolymph was very low in both fractions. Gram-positive bacteria were also affected by all larvae hemolymphs, immunized and naïve, in both fractions, and activities were dose dependent. Naïve larvae hemolymph was especially active against *M. luteus*.

Mazza *et al*. [14] studied the antimicrobial activity of methanol and pentane *R. ferrugineus* larvae cuticular surface extracts against Gram-positive, Gram-negative bacteria, and entomopathogenic fungi such as *Bacillus thuringiensis*, *E. coli*, *Bacillus bassiana*, and *Metarhizium anisopliae*. In addition, they found that methanol and pentane cuticular surface extracts of *R. ferrugineus* larvae. This study showed that methanolic extracts of large larvae surface components has inhibitory activity towards *B. subtilis*, *B. thuringiensis*, and *B. bassiana*, but no inhibitory activity toward *E. coli* and *M. anisopliae*. In addition, differences in antimicrobial activity were observed for different larval ages as well as different solvents used, where both methanol and pentane extracts of small larvae (10 small larvae) did not show any activity as well as all extracts using pentane as a solvent. This study also tested the anti-microbial capacity of larval hemolymph against *E. coli*, *Pseudomonas aeruginosa*, and *S. aureus*. No inhibitory activity was observed.

Sewify *et al*. [16] used the agar well diffusion method to test the *in vitro* microbial activities of different parts of *R. ferrugineus* digestive tract. The antimicrobial activity of extracts from various parts of the gastrointestinal tract was evaluated against Gram-positive bacteria (*E. faecalis* and *S. aureus*), Gram-negative bacteria (*E. coli* and *Klebsiella* spp.), *C. albicans*, and *Penicillium* spp. Foregut and hindgut extracts showed the most and least effective activity against the microbial species tested. The extracts exhibited the highest sensitivity towards Gram-positive bacteria and fungi, specifically *E. faecalis*, *S. aureus*, and *Penicillium* spp., as indicated by the biggest growth inhibition zones observed. The findings of this study demonstrate that gastrointestinal extracts possess significant antibacterial properties, suggesting their potential as a novel reservoir of AMPs.

Immune priming may increase the quality of insect immune responses, including antimicrobial responses. Mastore *et al*. [19], as explained above in this subchapter, and Shi *et al*. [43] and Zhang *et al*. [47] also conducted this study. Shi *et al*. [43] primed the *R. ferrugineus* immune system using phosphate-buffered saline (PBS) and *E. coli* and tested their phenoloxidase and antimicrobial activity against *E. coli* and *S. aureus* secondary infections. Immune priming with PBS resulted in lower phenoloxidase activity compared with *E. coli* immune-primed larvae and no increase in antimicrobial activity compared with non-immune primed larvae. The *E. coli* immune primed larvae showed a marked increase in both phenoloxidase and antimicrobial activity compared with non-immune primed larvae, and antimicrobial effects were seen in both *E. coli* and *S. aureus* incubated with hemolymph from immune primed larvae, with higher inhibition seen toward *E. coli*. This research also showed occurrences of mother-derived immune priming in offspring, where offspring from immune-primed larvae mothers showed a higher capability of phenoloxidase and antimicrobial activities when challenged with infection compared to non-immune primed parents and immune-primed fathers. A study by Zhang *et al*. [47] showed that immune priming of *R. ferrugineus* larvae with heat-killed *B. thuringiensis* caused a higher survival rate of this pest when dealing with secondary infection of the same bacteria, and higher clearance efficiency of *B. thuringiensis* and *Serratia marcescens*.

Muhammad *et al*. [44] showed that the intestinal microbiota of *R. ferrugineus* larvae promotes their immune responses through more effective antimicrobial activity and bacterial clearance against *E. coli* and *S. marcescens*, where conventionally reared (CR) larvae and germ-free (GF) larvae fed with its inherent microbiota from the gut homogenate (CR+BasSup) were more effective in both experiments compared to GF larvae. The colony-forming units (CFU) of *E. coli* extracted from the CR larvae hemocoel were 10-fold lower than that of *E. coli* in the hemocoel of GF larvae, and no statistical difference in CFUs was observed between the CR and CR+BasSup larvae, indicating that the reintroduction of its inherent microbiota can recover their pathogenic clearance capacity. *S. marcescens* challenged larvae showed a much higher mortality rate in GF larvae compared to CR and CR+BasSup larvae. These results indicate that the intestinal microbiota of *R. ferrugineus* promotes survival and pathogenic clearance.

### Mechanisms of action

The mechanism by which *R. ferrugineus* larvae work against microbes has not been studied in detail, although a number of actions may explain how these organisms fight them. The effect of AMPs present in *R. ferrugineus* larvae when faced with challenge or infection is one of the most frequently discussed [18, 19]. Knutelski *et al*. [18] and Mastore *et al*. [19] observed AMPs being present under conditions where this pest was challenged by bacteria and fungi. Attacin-B-like protein [18], which is rich in glycine [10] and one of the most studied AMPs, has been detected. It has isoforms whose sizes range from 20 to 23 kDa, and its activity is predominant toward Gram-negative bacteria [10]. It is likely that the activity of attacins is due to the presence of specific sections in their sequence that affect certain chemo-physical features, such as a positive charge or a high number of hydrophobic residues, that may potentially influence membrane interactions and subsequent antimicrobial activity [10]. Cecropin-A1-like peptide was another AMP found, cecropin is one of the most investigated AMPs in insects. Cecropins are helical linear peptides that lack cysteine and contain around 31–42 amino acids. They exert antimicrobial activities against a wide variety of microbes, including fungi, Gram-positive bacteria, and Gram-negative bacteria [18] by disrupting the microbial cell membranes [48]. Defensin has also been identified as a type of AMP in *R. ferrugineus*, which is a positive-charged peptide rich in cysteine with sizes of approximately 4 kDa that works against Gram-positive bacteria [49, 50]. A recombinant insect defensin impairs the permeability barrier of *M. luteus*’ cytoplasmic membrane, decreasing potassium and adenosine triphosphate (ATP) in the cytoplasm, depolarizing the inner membrane, and inhibiting respiration [51].

In a study by Mastore *et al*. [19], the AMP pool influenced bacterial cell walls by destabilizing and damaging the cell wall. Although some AMPs can also permeate the cell membrane and interact with the components present in the cytoplasm, AMPs are known to exert their effects towards the cell wall and outer membrane of bacteria. The effect of *R. ferrugineus* AMPs on cytoplasmic factors is not currently known; however, these studies show that one of the mechanisms of *R. ferrugineus* AMPs is directed toward the disruption of the bacterial cell wall.

Zhang *et al*. [47] explained that immune protection was enhanced when hemocyte proliferation occurred, which may be due to the phagocytic ability of hemocytes. They observed enhancement of phagocytic ability and pathogen clearance may be caused by the constant biosynthesis of serotonin within the hemolymph. In a previous study by Freire-Garabal *et al*. [52], serotonin increased phagocytic abilities in mouse peritoneal macrophages through 5-HT1A receptors.

The antimicrobial activity of *R. ferrugineus* may also be affected by modulation of immune-related genes in the intestine. As explained above, the intestinal microbiota plays an important role in the immune defense against *R. ferrugineus*, and depletion of the intestinal microbiota causes significantly lower activities against *E. coli* and *S. marcescens* [44]. A higher number of immune-related transcriptomes and genes were seen to be present in CR and CR+BasSup *R. ferrugineus* larvae, compared to larvae that had their intestinal microbiota removed [44]. These upregulated genes (some of which encode AMPs) play roles in immune detection, signal transduction and regulation (signaling pathways), immunological effector functions, and other related activities, and their presence enhances *R. ferrugineus* immune capabilities [44].

PO or phenoloxidase is an enzyme that has been highlighted in several studies Shi *et al*. [43] and Muhammad *et al*. [44] reported good PO activities after being challenged with *S. marcescens* and *E. coli* or PBS, respectively. Phenoloxidase generates indole molecules that are polymerized into melanin and is a key component in the immune system and melanogenesis of insects [53, 54]. The enzymatic reactions produce a number of intermediate substances, including reactive nitrogen intermediates, hydrogen peroxide, diphenols, quinones, and superoxides. These compounds play a crucial role in the defense mechanisms against both Gram-positive and Gram-negative bacteria, fungi, and viruses [54]. Enhancement of PO activity may be correlated with microbial clearance and survival rates of *R. ferrugineus* larvae [43, 44], suggesting that PO activity could be one of the mechanisms that support *R. ferrugineus* larvae’s immune response toward microbes.

## Comparison with other Antimicrobial Agents

### Overview of other broad-spectrum antibiotics and antimicrobial agents from insects

Broad-spectrum antibiotics have been developed for a long time and are efficacious towards Gram-negative and Gram-positive bacteria [55, 56]. They are often used in cases where the pathogenic agent is unknown or when multiple pathogenic agents are present [57]. Amoxicillin-clavulanate, ampicillin, trimethoprim/sulfamethoxazole, and azithromycin are some of the most well-known and used antibiotics. Although most of these agents show great efficacy against a diverse array of bacteria, there is a rising trend of antibacterial resistance that causes the birth of novel bacterial strains that are not affected by these antibiotics [58].

Insects are a vast reservoir of physiologically active chemicals, and hence, serve as a source of natural products that have found diverse applications in traditional medicine for many years and continue to be essential sources of therapeutic substances [12]. Insects constitute a large group of organisms in the animal kingdom, and their rich biodiversity makes them a promising source of alternative antibiotics. Insect antimicrobial substances, such as AMPs, typically exhibit a broad spectrum of activity and possess the ability to circumvent the resistance mechanisms commonly associated with conventional antibiotics [12, 49]. It could solve the problem of multidrug resistance of a wide range of microorganisms to conventional antibiotics.

In addition to *R. ferrugineus*, the utilization of insect larvae as a potential antimicrobial source has been studied in many other organisms, including *Hermetia illucens*, *Lucilia sericata*, *Musca domestica*, *Chrysomya megacephala*, *Tenebrio molitor*, *Sarconesiopsis magellanica*, and *Scarabaeus sacer*. Extracts from all these insects demonstrated significant antimicrobial activity against Gram-positive and Gram-negative bacterial strains [59–65].

### Comparison of the efficacy of *R. ferrugineus* larvae with other antimicrobial agents

Thus far, *R. ferrugineus* larvae have not been studied clinically for the purpose of development of an antimicrobial agent. However, as explained above, studies have observed and tested its antimicrobial activities and show that *R. ferrugineus* possesses the potential of being developed into a broad-spectrum antimicrobial agent [14, 17–19, 43, 44, 47]. The antimicrobial capacity of *R. ferrugineus* larvae has never been directly compared with other antimicrobial agents; therefore, it is difficult to directly compare the efficacy of *R. ferrugineus* against other antimicrobial agents. Commercial antibiotics are available with a wide range of functions against different bacteria.

Amoxicillin-clavulanate is an antibiotic with broad-spectrum activity against a variety of Gram-positive and Gram-negative bacteria and possesses lactamase activity [66]. Amoxicillin is originally a narrow-spectrum antibiotic that is efficacious against Gram-positive bacteria and only a few Gram-negative bacteria. However, with the incorporation of clavulanic acid, β-lactamase-producing isolates and other bacterial species are added to the spectrum of their efficacy [66, 67]. This antibiotic is commonly used for treating otitis media, pharyngitis, tonsilitis, respiratory tract infections, urinary tract infections (UTIs), tuberculosis, pelvic inflammatory disease, and bite wounds [68]. Ampicillin is another broad-spectrum antibiotic that belongs to the aminopenicillin class of the penicillin family [69] and is a lactam antibiotic. This antibiotic is used to treat endocarditis, meningitis, central nervous system infections, respiratory tract infections, septicemia, UTIs, pertussis, salmonella, shigella, eikenella, and listeria infections [70]. Trimethoprim/sulfamethoxazole, a broad-spectrum antibiotic consisting of one part trimethoprim to five parts sulfamethoxazole [71], is also on the World Health Organization (WHO) list of essential medicines [72]. Sulfamethoxazole is a sulphonamide, whereas trimethoprim is a diaminopyrimidine. When alone, sulfamethoxazole exhibits bacteriostatic activity, but when combined, it may exert bactericidal anti-folate effect [73]. This medication is used to treat travelers’ diarrhea, otitis media, acute infective exacerbation of chronic bronchitis, UTIs, pneumonia from *Pneumocystis jirovecii* or *Pneumocystis carinii*, and shigellosis [73]. Azithromycin is a broad-spectrum antimicrobial that is included in the WHO list of essential medicines [72]. It belongs to the macrolide class of antimicrobials and is potent against a number of Gram-negative and Gram-positive bacteria [74–76]. This antibiotic is oftentimes used for the therapeutic management of cutaneous or skin infections and its associated conditions such as acne vulgaris [77–79] as well as sexually transmitted infections (STIs), such as gonorrhea, caused by the pathogenic Gram-negative bacteria *Neisseria gonorrhoeae* [80, 81], either on its own or in combination with other medications such as gemifloxacin, gentamicin, and extended-spectrum cephalosporins [82–84]. Cefixime (as an alternative to ceftriaxone) is another well-known antibiotic that is utilized for the treatment of gonorrhea and is also efficacious against a vast array of both Gram-positive and Gram-negative bacteria, such as all strains of group A *Streptococci*, *Haemophilus influenzae*, *E. coli*, and *Moraxella catarrhalis*, regardless of their β-lactamase production status, as well as strains of pneumococci that are penicillin sensitive [85–87]. Clindamycin is a medication used for the treatment of skin and skin structure infections as well as intra-abdominal infections, lower respiratory, gynecological, bone and joint infections, and septicemia [88]. It is also known to be effective against Methicillin-resistant *S. aureus* (MRSA) infections but has recently been shown to be ineffective due to inducible resistance [89, 90].

Although these antibiotics are some of the most used antibiotics, they do not come without the risk of adverse effects, such as gastrointestinal symptoms in all six medications, dermatologic effects in amoxicillin-clavulanate and clindamycin, seizures in ampicillin and amoxicillin-clavulanate, hepatotoxicity in ampicillin and azithromycin, and headache and dizziness in ampicillin and trimethoprim/sulfamethoxazole [67, 69, 73, 74, 88, 91].

An alternative for the development of antimicrobials capable of combating antibiotic-resistant bacterial strains as well as fungicidal effects is the use of insect sources. *H. illucens* has also been tested for its antimicrobial activity. Extracts of this pest have been immunized against several bacterial strains, and it has been concluded that the *Lactobacillus casei*-immunized *H. illucens* extract possesses potent antibacterial properties and serves as a natural preservative that mitigates the risk of contamination by various *Salmonella* species [59]. Another pest of interest is *L. sericata*, which in a research by Amer *et al*. [64], its maggot extracts showed great activity against Gram-negative bacteria (*Klebsiella pneumoniae*, *E. coli*, and *P. aeruginosa*), Gram-positive bacteria (*Streptococcus pyogenes*, *S. aureus*, and *B. subtilis*), and several fungi (*Geotricum candidum*, *Aspergillus flavus*, *Aspergillus fumigatus*, *Penicillium* spp.) [64]. The larvae of *M. domestica* and *C. megacephala* were studied in the same research by Sahalan *et al*. [65] for their antimicrobial activities. *M. domestica* larvae exhibited antibacterial properties through the lysis of *B. subtilis* and two Gram-negative bacteria, *E. coli* and *P. aeruginosa*, whereas *C. megacephala* showed bactericidal effect against *B. subtilis* and *S. aureus*, and no activity was seen against Gram-negative bacteria. Hwang *et al*. [62] observed the antimicrobial activities of peptide-overexpressed *T. molitor* larvae extract for use as a natural preservative and showed that these extracts possess concentration-dependent inhibitory activity against foodborne bacteria like *Bacillus cereus*, *S. aureus*, and *E. coli* as well as against harmful fungi like *Aspergillus parasiticus*, *A. flavus*, and *Pichia anomala*. Sarconesin, an excretion and secretion product of *S. magellanica* exhibited significant inhibitory efficacy against Gram-negative (*E. coli*, *Salmonella enterica*, and *P. aeruginosa*) and Gram-positive (*S. aureus*, *Staphylococcus epidermidis*, and *M. luteus*) bacteria [63]. Mohamed *et al*. [8] tested the antimicrobial activity of *S. sacer* hemolymph against fungi (*A. fumigatus* and *C. albicans*), Gram-positive bacteria (*S. aureus* and *B. subtilis*), and Gram-negative bacteria (*E. coli* and *Enterobacter cloacae*), where the hemolymph exhibited the greatest antibacterial action against Gram-negative bacteria (*E. cloacae* more than *E. coli*), followed by Gram-positive bacteria *B. subtilis* more than *S.aureus*). These studies show that potential antimicrobial agents sourced from insects can act against a wide range of microbes and can compete with conventional broad-spectrum antibiotics, with the potential to circumvent the antibiotic resistance of a number of microorganisms [8, 59, 61–65].

### Advantages and Limitations of using *R. ferrugineus* Larvae

The main advantage of using *R. ferrugineus* larvae as an antimicrobial agent is its broad-spectrum antimicrobial activity. *R. ferrugineus* larvae show remarkable activity against several Gram-negative bacteria, Gram-positive bacteria, and fungi. This suggests that *R. ferrugineus* possess broad-spectrum antimicrobial properties; however, further research is needed to confirm this conclusion [14, 17–19, 43, 44, 47]. Another advantage of this pest is that it is a natural source of AMPs. Knutelski *et al*. [18] observed that *R. ferrugineus*’ hemolymph consists of several different AMPs that have been shown to have great antimicrobial activity. AMPs are a promising alternative to conventional antibiotics because they possess a broad spectrum of activity and are less likely to induce resistance [12, 49]. Furthermore, the use of biocontrol agents, such as gut bacteria from *R. ferrugineus*, as suggested in a study by Liu *et al*. [15], for pest control has been suggested as a low-toxicity alternative to chemical pesticides, which may suggest that the use of *R. ferrugineus* larvae as an antimicrobial agent may also have low toxicity. In addition to the advantages of its use as an antimicrobial agent, it is also advantageous to utilize these pests for beneficial purposes. The use of *R. ferrugineus* larvae as an antimicrobial agent could be a sustainable solution to pest control, where instead of using chemical pesticides that can be harmful to the environment and other organisms, utilizing the larvae of *R. ferrugineus* could provide a natural and eco-friendly alternative solution for this issue. In addition, the use of this pest as an antimicrobial agent could provide economic benefits to communities that are affected by it as the larvae could be utilized for a beneficial purpose rather than being discarded.

Although this finding has a number of advantages, it does not have its own limitations. These limitations stem from a lack of studies using this agent as an antimicrobial agent. Analyses of its antimicrobial activity have been carried out several times [14, 17–19, 43, 44, 47]; however, more *in vitro* and *in vivo* investigations, together with clinical trials, need to be conducted in order to fully understand the antimicrobial efficacy of this drug. The antimicrobial activity of *R. ferrugineus* larvae has also been seen to show different results in different studies. For example, Mazza *et al*. [14] showed that *R. ferrugineus* was not efficacious against Gram-negative bacteria, whereas Sewify *et al*. [17], Knutelski *et al*. [18], Mastore *et al*. [19], Shi *et al*. [43], and Muhammad *et al*. [44] showed the opposite result. However, this may be caused by variations in the concentration of the pest extracts used in the experiments. One limitation is the lack of standardization. Consistent quality of *R. ferrugineus* larvae has not yet been guaranteed or verified, irrespective of whether the entire larva is used for extracts or only a portion of the body.

Despite the abundance of benefits, additional research is required to fully comprehend the antimicrobial capabilities and limitations of this pest so that it can be utilized to its fullest potential.

## Safety and Toxicity Considerations

### Examination of the safety profile of *R. ferrugineus* larvae and assessment of potential toxicological effects and allergic reactions

Although *R. ferrugineus* larvae are commonly consumed in many regions of Asia, there is still insufficient data on their clinical safety. Instead of assessing their safety profile and toxicity, the majority of available research focuses on their destructive nature as pests or their potential as food sources. Kavle *et al*. [27] investigated the potential health implications of *R. ferrugineus* larvae consumption and found that *R. ferrugineus* larvae are rich in essential minerals and contain trace amounts of heavy metals that are below acceptable toxicity limits. However, it did not contain polyunsaturated fatty acids and was rich in saturated fatty acids. Overall, it is a valuable and safe food product; however, its fatty acid content presents some personal considerations. Mastore *et al*. [19] investigated the hemolytic abilities of *R. ferrugineus*’ hemolymph against human red blood cells (RBCs) and discovered that whole hemolymph from the naïve larvae caused 5.48% hemolysis of RBCs compared to a control of 100% lysis by incubation with 0.02% Triton X-100. It was also seen that <30 kDa fractions of the naïve hemolymph caused lower lysis (5.12%), followed by <30 kDa fractions of immunized (with Gram-positive and Gram-negative bacteria) hemolymph (4.88%), and then by an astounding no lysis observed in incubation of RBCs with <10 kDa immunized hemolymph fraction.

Although it appears to be a relatively safe insect for use as an antimicrobial agent in *in vitro* conditions, it is unknown how its effects will change once it has been transformed into an antimicrobial agent and when it is ingested orally, topically, or through other routes in humans [92]. However, the pharmacodynamics of this medicine remain largely unknown. There is a need to carry out further studies of the substances present in *R. ferrugineus*, taking into account the allergenic risk of each of them. Barre *et al*. [93] studied the presence of Immunoglobulin (Ig) E-binding cross-reactive allergens in a number of edible insects such as *R. ferrugineus*. IgE is an antibody that is widely associated with various allergies and is known for its function in mediating allergic reactions [94]. Barre *et al*. [93] detected acidic ribosomal protein, actin−α, arginine kinase, chemosensory protein, cytochrome C, fatty acid-binding protein, odorant-binding protein, fructose-1,6-biphosphate aldolase, hexamerin, glyceraldehyde-3-phosphate dehydrogenase, α-myosin, larval cuticle protein, and tropomyosin 1. The presence of chitin in their exoskeleton may also elicit an allergic response in several individuals with minute quantities of bacterial chitinolytic enzyme in their gastrointestinal system [95].

### Comparison of *R. ferrugineus* safety profile to traditional antibiotics

As previously explained by Yew and Kok [96] only allergic reactions and a case of Takotsubo cardiomyopathy have been observed in *R. ferrugineus* larvae to date; no other adverse effects have been reported to date. The lack of information may be due to the fact that it has not yet been evaluated in clinical settings, and its antimicrobial extracts may have distinct effects compared to oral consumption as a food source [92]. Conventional antibiotics have been associated with a variety of side effects, including headaches, dermatologic effects, dizziness, gastrointestinal syndromes, and even seizures [67, 69, 73, 74, 88, 91]. However, they have been extensively studied and have a well-established safety profile if they are used under the right conditions and at the right dosage and under medical supervision. However, *R. ferrugineus* is a safe, insect-derived, and natural antimicrobial agent with a broad spectrum and a low likelihood of inducing antibiotic resistance [8, 12, 49]. However, the low toxicity of *R. ferrugineus* [19, 29] supports the notion that it is a safe, insect-derived, and natural antimicrobial agent with a broad spectrum and low likelihood of inducing antibiotic resistance [8, 12, 49].

The safety profile of *R. ferrugineus* larvae as an antimicrobial agent is not as well-studied compared to conventional antibiotics. Despite its antibacterial properties [14, 17–19, 43, 44, 47], further research is needed to evaluate its safety and possible adverse effects. Traditional antibiotics, on the other hand, have a long history of use and a well-documented safety profile, even though they are not risk-free.

## Potential Clinical Applications

### Overview of the potential clinical applications of *R. ferrugineus* larvae as an antimicrobial agent

*R. ferrugineus* has demonstrated antimicrobial activity against an array of microbes and is thought to have the potential to become an antimicrobial agent [14, 17–19, 43, 44, 47]. AMPs and digestive system extracts [18, 19, 44] are some aspects of this pest that have potential for this utilization. Since there have been no clinical trials or use of their AMPs or extracts, it is not yet known the exact product for the use of the pest. In the development of novel antimicrobial agents, their extracts may serve either as components or as whole extracts, depending on the microbes against which they are active. Several parts of the larvae body that have potential to be utilized in clinical applications are alimentary canal extracts (including the gut) and their hemolymph due to the presence of AMPs.[14, 17–19, 43, 44, 47].

Clinical use of *R. ferrugineus* larvae can be based on their activity against the microbes. *E. coli*, a Gram-negative bacterium frequently encountered in the lower gastrointestinal tract of endothermic species such as humans and animals [17–19, 43, 44, 97, 98], is one of these microbes. Most strains of this bacterial species are harmless and may even be beneficial, although some strains may act as pathogens causing diseases, such as different types of diarrheal illnesses, uncomplicated cystitis, pneumonia, bacteremia, and spontaneous bacterial peritonitis [99]. The intervention of diseases caused by *E. coli* could be a potential clinical use of *R. ferrugineus* larvae extracts [17–19, 43, 44], most probably through oral routes of administration.

*M. luteus*, a Gram-positive bacterium that is generally harmless and found in many places including the human skin, may turn into an opportunistic pathogen in immunocompromised people, where it causes nosocomial infections [19, 100]. In recent years, there have been reports suggesting that this particular bacterium may cause various illnesses, including bacteremia, native valve endocarditis, hepatic and brain abscess, and septic arthritis, particularly in patients with compromised immune systems, as well as BSI in immunocompromised patients [100]. *R. ferrugineus* larvae extracts may be a potential antimicrobial agent as a means of therapeutic against the various diseases caused by this bacterium [19, 100].

*S. aureus* is another Gram-positive bacterium affected by *R. ferrugineus* extracts [17, 43]. This bacterium is usually found in the skin of humans and may be pathogenic under certain circumstances, causing skin infections, including abscesses, boils, furuncles, impetigo, surgical site infections, and cellulitis [101]. Several strains of this bacteria, such as MRSA, are methicillin resistant and are currently on the rise [102]. *R. ferrugineus* extracts possess the potential to be utilized as a broad-spectrum antimicrobial agent that is efficacious against different strains of *S. aureus* [17, 43], including MRSA.

A Gram-negative bacterium whose activity is inhibited by *R. ferrugineus* extracts is *Pseudomonas* spp., which opportunistically causes diseases in plants and animals and is a multi-drug resistant pathogen [19, 103]. *Pseudomonas* spp. have been identified as causative agents of severe infections, such as meningitis, septicemia, malignant external otitis, endocarditis, endophthalmitis, and pneumonia, as well as skin and soft-tissue infections [104]. This bacterium is known to be resistant toward a wide array of antibiotics and may exhibit additional resistance subsequent to an ineffective course of treatment, and often used as a model bacterium in studies of bacterial social traits and virulence [103]. *R. ferrugineus* may potentially act as an intervention of this bacteria’s activity due to its broad-spectrum activity and proof of efficacy against *Pseudomonas* spp. [19].

Another Gram-negative bacterium that has the potential to be affected by *R. ferrugineus* larvae extract is *N. gonorrhoeae*, the bacterium that causes the gonorrhea disease or STI [81]. The Gram-negative bacteria that have already shown to be inhibited by *R. ferrugineus* larvae extract, namely, *E. coli* and *Pseudomonas* spp., have several similarities, such as high structural similarity between a key enzyme in amino acid and metabolite biosynthesis in *Pseudomonas* spp. and *N. gonorrhoeae*, aspartate-semialdehyde dehydrogenase [105]. *N. gonorrhoeae* possesses outer membrane vesicles that contain β-lactamases which aids in antibiotic resistance, similar to *Pseudomonas* spp. [106], and *N. gonorrhoeae* produces chromosomal DNA through a novel Type IV secretion system, where some of its genes are similar to those of *E. coli* [107]. This shows that even though *R. ferrugineus* larvae extracts’ antimicrobial activity against *N. gonorrhoeae* has not been tested yet, the similarities of this bacterium with other Gram-negative bacteria that has been inhibited by *R. ferrugineus* larvae support the notion that it may show the same potent activity against this bacterium [105–107].

Besides being effective against bacteria, *R. ferrugineus* has also shown activity against *C. albicans*, which is an opportunistic pathogenic fungus in immunocompromised patients [17, 108]. It is a common member of the human gastrointestinal system and mouth, and a major contributing fungi to candidiasis and causes 30–40% mortality in candidemia [109, 110]. Just like antibiotic resistance, antifungal resistance also occurs which gives way for the potential utilization of *R. ferrugineus* larval extracts.

### Challenges and limitations of clinical applications

Clinical applications seem like the best next step for the utilization of *R. ferrugineus*’ antimicrobial properties, challenges are still abundant. One challenge is once again the limited number of studies, especially on clinical trials in human or *in vitro* using human-derived cells or tissue. These types of studies may provide a solid foundation for the use of this insect’s extracts as a medically valuable antimicrobial substance and will likely address questions about its efficacy and safety profile when administered to humans. Its use in clinical settings may also be hindered by regulatory bodies, requiring extensive testing and evaluation before approving its use. The consistent quality and uniformity of *R. ferrugineus* larvae in a large-scale setting also needs to be taken into consideration, where standardization and quality control measures need to be established to ensure reliable and reproducible results, as well as standardization of extract concentration, extract solvent, and dosage. Another challenge is the potential of allergic reactions to this pest, where the presence of various IgE-binding cross reactive allergens, as well as the presence of chitin on their exoskeleton may work as factors that cause allergic reactions toward this pest, whether through uptake as an antimicrobial agent or as consumed food [93, 95], and in a report by Yew and Kok [96], a middle-aged man had a reaction similar to anaphylaxis after eating 20 cooked and ready to consume *R. ferrugineus* larvae for thefirst time. Besides this, consumption of *R. ferrugineus* larvae in Indonesia also commonly comes with a warning of anaphylaxis-like reactions, especially itchiness, although there have been no known reports of critical medical reactions. Even so, this aspect needs to be thoroughly investigated to assess the potential risks associated with its use.

### Implications for future research

The antimicrobial potential of *R. ferrugineus* larvae has been exhibited in a number of research, but once again, before turning it into an actual antimicrobial agent, more specific studies need to be conducted and this review suggests that future research should include studies about standardization of larvae age used as antimicrobial agents. The differences in pest age show different antimicrobial capacities [14]; hence, a more thorough examination of the optimum larvae age would help get the most out of their antimicrobial properties. The differences in food sources [28, 30] exhibited slightly different antimicrobial activities. The optimum food source of *R. ferrugineus* larvae must be considered to be able to breed and select the best larvae for this purpose. Standardization of extraction method is also a factor to consider, as different methods of extraction and different solvents utilized usually produce varying qualities and concentrations of extract, and some solvents used might even kill the tested microbes and alleviate the activity of the pest’s extract [14]. Establishing standardized protocols for the extraction and preparation of *R. ferrugineus* larvae extracts is essential to ensure consistent quality and potency, and implementing quality control measures will help maintain batch-to-batch consistency and ensure reliable results. Exploring different formulations and delivery methods as well for *R. ferrugineus* larvae extracts can enhance their stability, bioavailability, and ease of administration. This may involve developing topical formulations, oral formulations, or incorporating the extracts into existing antimicrobial products.

Further investigation and characterization of its antimicrobial compounds is needed to understand the specific mechanisms by which *R. ferrugineus* larvae exert their antimicrobial activity. Some of the AMPs identified in *R. ferrugineus* mechanisms have been explained [18], but more characterizations of other antimicrobial properties of this pest needs to be conducted. Elucidating the mode of action can provide information about the potential targets and pathways involved, aiding in the development of more targeted and effective treatments.

A method to improve their antimicrobial activity is through immune priming, as exhibited in several studies. This method of insect preparation by inducing antimicrobial action through immunization with bacteria causes an increase in antimicrobial activity (whether through inhibiting or killing) when dealt with infection [19, 43, 47]. Another way is through investigating the potential synergistic effects of *R. ferrugineus* larvae extracts with existing antimicrobial agents, which could lead to the development of combination therapies. This approach may enhance antimicrobial efficacy, reduce the risk of resistance, and broaden the spectrum of activity.

Besides bettering the quality of the product, safety, and toxicity is also a very important factor to consider, as well as how it will fair in clinical settings and long-term efficacy. Conducting comprehensive safety and toxicity studies is crucial to assess the potential risks associated with the use of *R. ferrugineus* larvae as antimicrobial agents. This includes evaluating potential allergenicity, determining the maximum tolerated dose, and assessing any adverse effects on human cells or tissues. Even though some of these topics have been previously discussed by Mastore *et al*. [19], Köhler *et al*. [95], information is still at a minimum and more studies need to be done to assure the safety profile and tolerability of this pest’s extracts as antimicrobial agents. Assessing the long-term safety of using *R. ferrugineus* larvae extracts is also important in identifying any potential cumulative effects or resistance development. Long-term studies can provide insights into the sustainability and durability of their antimicrobial activity, and some reassurance about the broad-spectrum antimicrobial properties. Clinical efficacy and safety evaluations of this potential antimicrobial agent needs to involve conducting well-designed clinical trials to assess the effectiveness of these extracts against specific bacterial and fungal infections, comparing them with standard antimicrobial agents.

## Conclusion

Antimicrobial resistance is a developing concern, as the proliferation of microorganisms resistant to antibiotics makes the management of infections more troublesome. Eggs, larvae, and adults of *R. ferrugineus* can inhibit Gram-negative bacteria, Gram-positive bacteria, and fungi, according to studies. Due to its broad-spectrum antimicrobial activity against numerous bacteria and fungi, *R. ferrugineus* larvae has shown promise as an antimicrobial agent with potential applications in treating bacterial infections and preventing antibiotic resistance. The larvae’s AMPs and digestive system extracts have the greatest potential for clinical applications, although the precise product for the utilization of this pest has not yet been determined. The larvae’s extracts can be used as components or whole extracts, with the type of medication, dosage, and route of administration being dependent on the microbes they are efficacious against, such as *E. coli*, *M. luteus*, *S. aureus*, *Pseudomonas* spp., *C. albicans*, and potentially efficacious against, such as *N. gonorrhoeae*.

The gastrointestinal microbiota of *R. ferrugineus* promotes the growth and development of the organism. *In vitro* research has revealed that *R. ferrugineus* larvae possess an arsenal of AMPs that assist in their survival in an environment favorable to bacterial growth. The mechanism of action of *R. ferrugineus* larvae against microorganisms has not been investigated in detail; however, multiple actions may explain how they combat these organisms. The effect of AMPs present in the larvae in response to a challenge or infection is one of the most discussed mechanisms. The modulation of immune-related genes in the intestine and the phagocytic ability of its hemocytes may also influence the antimicrobial activity of *R. ferrugineus* larvae, with an increase in PO or phenoloxidase activity possibly correlated with microbial clearance and survival rates of *R. ferrugineus* larvae.

Clinical applications of the antimicrobial activity of *R. ferrugineus* larvae are promising, but challenges remain, including insufficient studies, regulatory bodies, and the possibility of allergic reactions. Standardization and quality control measures are essential for achieving uniformity and consistency on a large scale. The presence of various IgE-binding cross-reactive allergens and chitin on the larvae’s exoskeleton may also contribute to allergic reactions. The safety and toxicity of *R. ferrugineus* larvae extracts, as well as their long-term efficacy, are crucial considerations. To assess potential hazards, allergenicity, maximum tolerated dose, and adverse effects on human cells or tissues, it is essential to conduct exhaustive safety and toxicity studies.

## Implications for future research

Future research should focus on standardizing the age of larvae used as antimicrobial agents, considering disparities in the age of pests and their food sources. For consistent quality and potency, standardization of extraction techniques and solvents is crucial. Exploring alternative formulations and delivery methods can also improve stability, bioavailability, and administration convenience. To fully understand the precise mechanisms by which antimicrobial compounds from *R. ferrugineus* larvae exert their antimicrobial activity, further investigation and characterization are required. Immune priming, a method of insect preparation that induces antimicrobial action by immunizing insects with bacteria or other substances, can enhance antimicrobial activity. Combination therapies with existing antimicrobial agents may improve antimicrobial efficacy, reduce the risk of resistance, and expand the spectrum of activity.

Understanding the mechanisms of action, conducting safety and efficacy evaluations, developing formulations, assuring standardization, and exploring immune priming and combination therapies should be the focus of future research on *R. ferrugineus* as antimicrobial agents. These findings will aid in the development of *R. ferrugineus* larvae extracts as a safe and effective antimicrobial treatment.

## Authors’ Contributions

NJN and TET: Conceptualization, supervision, and funding acquisition. NJN, SSH, and TET: Methodology, software, and project administration. BJK: Validation and formal analysis. NJN, BJK, and TET: Resources. NJN, BJK, TSK, and TET: Data curation. NJN, TSK, and TET: Writing-original draft preparation and writing-review and editing. NJN and BJK: Visualization. All authors have read, reviewed, and approved the final manuscript.
